# Partial Discharge in Nanofluid Insulation Material with Conductive and Semiconductive Nanoparticles

**DOI:** 10.3390/ma12050816

**Published:** 2019-03-11

**Authors:** Mohamad Zul Hilmey Makmud, Hazlee Azil Illias, Ching Yern Chee, Sameh Ziad Ahmad Dabbak

**Affiliations:** 1 UM High Voltage Laboratory (UMHVL), Department of Electrical Engineering, Faculty of Engineering, University of Malaya, Kuala Lumpur 50603, Malaysia; al_dabak@hotmail.com; 2 Complex of Science and Technology, Faculty of Science and Natural Resources, Universiti Malaysia Sabah, Kota Kinabalu 88400, Malaysia; 3 Department of Chemical Engineering, Faculty of Engineering, University of Malaya, Kuala Lumpur 50603, Malaysia; chingyc@um.edu.my

**Keywords:** nanofluid, partial discharge, high voltage, electrical insulation

## Abstract

This study provides a thorough investigation of partial discharge (PD) activities in nanofluid insulation material consisting of different types of nanoparticles, which are conductive and semiconductive when subjected to high voltage stress is presented. Nanofluids have become a topic of interest because they can be an alternative to liquid insulation in electrical apparatus due to their promising dielectric strength and cooling ability. However, during in-service operation, PDs can occur between conductors in the insulation system. Therefore, this study presents the behavior of PDs within nanofluid dielectric materials consisting of conductive and semiconductive nanoparticles. The results show that there is an improvement in the PD resistance and a reduction in the tan delta of nanofluids at power frequency after the incorporation of conductive or semiconductive nanoparticles in the nanofluid oil. However, the most suitable concentration of conductive and semiconductive nanoparticles in the base fluid was found to be, respectively, 0.01 g/L and 1.0 g/L at PD inception and PD steady-state conditions. The clustering of nanoparticles in a nanofluid suspension due to PD activities is also discussed in this study.

## 1. Introduction

A high voltage transformer is a key component in an electrical power system network. It plays a vital role in each division of power system grid, which are generation, transmission, and distribution. The majority of high voltage transformers depends on liquid dielectric as an insulation material. In recent years, advances in nanotechnology have led to the formation of a remarkable new material, nanofluid insulation, which is produced through the addition of nanoparticles to a base insulating liquid. Insulation-based-nanofluid has been shown to have high dielectric strength and significant improvement in cooling ability compared to the base materials [1,2]. However, the majority of these nanofluid insulations are not yet commercially available due to certain problematic factors that require further investigation [3]—for instance, the stability of nanoparticles when being dispersed into a base fluid, interaction with the magnetic field in a transformer, and the effect of high electric field on the performance of nanofluids over time. 

Therefore, one of the significant factors to be considered is the influence of the electrical properties of nanoparticles on partial discharge (PD) behavior in nanofluids. Generally, PD can occur inside a liquid insulation system when the electric field is enhanced in a localized area between the dielectric material and the conductor. This leads to the acceleration of thermal aging and streamer breakdown development [4]. Herchl et al. carried out PD measurements in a magnetic-based nanofluid insulation [5]. They found that PDs contributed to losses and caused a progressive degradation of the nanofluid. In nanodielectrics, it is commonly accepted that adding inorganic nanoparticles into a polymer matrix is an effective method to enhance PD resistance of solid insulation [6,7]. Nanoparticles act as a systemic barrier to prevent the insulation from high-energy bombardment activities, such as PDs [8]. Moreover, in the nanofluid, the species on the nanoparticle’s surface affected the discharge dynamics. As reported in [9,10], silica nanoparticles can adsorb water, acid, or impurities, leading to a reduction in PD activities, as well as improve the breakdown voltage of the insulation oil.

Recent research has postulated that the enhancement of dielectric strength of nanofluid is due to electron trapping on the surface of the nanoparticles [11,12,13]. It has been suggested that the trapping mechanisms of nanoparticles are dependent on charge induction in conductive nanoparticles or by polarization in semiconductive and insulating nanoparticles. However, there are no comparative studies on the influence of the electrical properties of different nanoparticles, such as conductivity and permittivity, on PD behaviour in nanofluid insulation material. Therefore, this study provides an opportunity to gain a better understanding on the matter by comparing PD behaviour in nanofluids, which consist of conductive and semi-conductive nanoparticles dispersed in natural ester oil.

In this study, at the beginning, tan delta measurements were obtained in order to assess the initial performance of the base oil after the incorporation of different types of nanoparticles. Then, the influence of the electrical properties of these nanoparticles on PD activities was studied under two conditions: inception and steady-state conditions. PDs data at the inception condition were used to calculate the probability of PD occurrences using Weibull distribution. The results from steady-state condition were statistically compared in term of PD characteristics, which include PD events, total PD charges, maximum charge magnitude, and PD patterns.

## 2. Materials and Methods 

### 2.1. Sample Preparation

Highly-refined palm oil (PO) was used as natural ester-based insulating fluid (NE) [14]. Two different types of nanoparticles—Iron (III) oxide (Fe_3_O_4_) ≥ 97% and Titanium dioxide (TiO_2_) ≥ 99.5%—were applied to prepare the samples of natural ester with conductive nanoparticles (CNF) and natural ester with semiconductive nanoparticles (SNF), respectively. The base fluid and nanoparticles, which were commercially obtained, were kept for 24 h at the elevated temperature (80 °C for the base fluid and 300 °C for nanoparticles) and under vacuum conditions (90 kPa) to remove moisture and impurities. The average of water content for the base fluid was recorded as 110.63 ppm using Metrohm Karl Fischer (KF) Coulometer. 

Six samples of natural ester-based nanofluids were prepared by ultrasonication for 30 min at room temperature using an ultrasonic bath (output power = 120 W, frequency = 40 kHz). The abbreviation for each sample is shown in Table 1. The aggregate distribution size of the nanofluids is measured at ≤ 100 nm using dynamic light scattering (DLS) with a Malvern Zetasizer (Malvern, UK). All samples are visually stable after 24 h as reported in the previous study [15]. 

### 2.2. Tan Delta Measurement Method

Tan delta was measured to evaluate the nanofluid insulation quality with the existence of nanoparticles by the ratio between dielectric loss *ε*” and dielectric constant *ε*’. Electrochemical Impedance Spectroscopy (EIS) method was used by applying a 1 V peak-to-peak voltage at the frequency range between 0.1 Hz and 0.1 MHz at room temperature conditions. The setup consisted of two cylindrical copper plates (working and ground electrodes) with a diameter of 25 mm and 100 μm gap distance. Both electrodes were fully immersed in the fluid under test. Tan delta was measured before each sample was stressed with the applied voltage for PD measurement. The tan delta was obtained from the dielectric loss and dielectric constant values by applying

(1) $$\tan\delta =\frac{\varepsilon^{\prime \prime }}{\varepsilon^{\prime }}$$

### 2.3. PD Measurement Method

**Figure 1:**
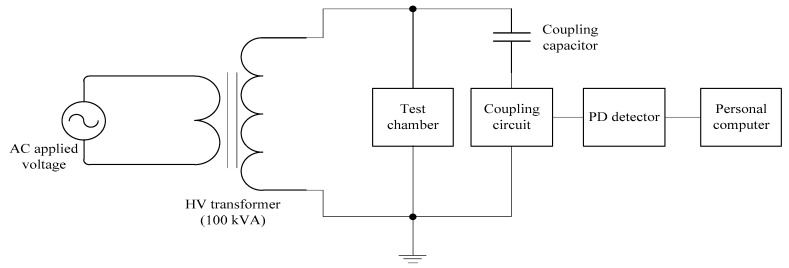
Partial discharge (PD) measurement setup.

The Mtronix MPD 600 setup manufactured by Omicron (Germany) was used for PD measurement in conjunction with a high voltage generation kit. The schematic diagram of the PD measurement system is shown in Figure 1. The transformer has a rating of 0.22/100 kV, 100 kVA. The PD signals measured by the MPD 600 were in current pulses across the nanofluid sample between the needle and plane electrodes. When PDs occur, the coupling capacitor transfers equivalent charges to the test object to preserve the voltage drop across the electrodes. Voltage pulses in the nanosecond range are detected by the coupling device and the PD detector, which are transferred through fibre optics to a personal computer (PC).

**Figure 2:**
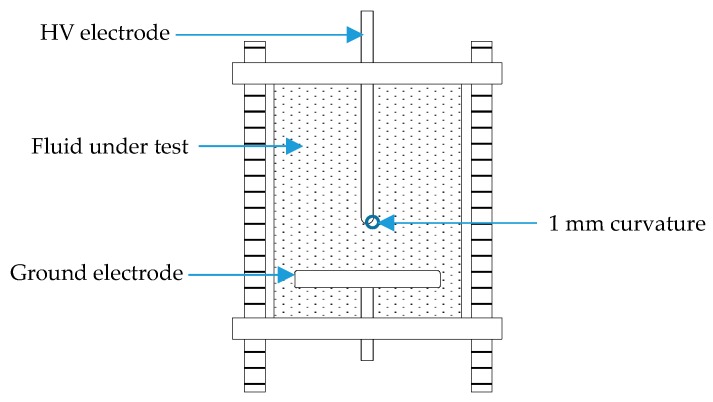
Schematic diagram of the test chamber for PD measurement.

Figure 2 shows a schematic diagram of the test chamber used for PD measurement in a nanofluid sample. The self-fabricated chamber was adopted from IEC 60343 configuration which consists of two conductors; high voltage and ground stainless steel electrodes. The rod electrode has a 1 mm curvature radius and 10 mm gap distance to the plane electrode. The edges of the test chambers were sealed with silicone glue to prevent the fluid under test from spilling out. This setup allows PDs to occur without causing any breakdown. The nanofluid samples of 400 mL volume in test chamber were stressed by being subjected to 50 Hz sinusoidal applied voltage. The voltage was controlled at a rate of rise of 1 kV/s. The PD measurement consists of two conditions:

- Inception condition: Measurement was taken immediately after the first PD occurrence. The applied voltage *U_app_* was ramped-up from 0 to 25 kV.
- Steady-state condition: Measurement was taken after 30 min of the applied voltage. The applied voltage *U_app_* was fixed at 26, 28, and 30 kV.

## 3. Results

### 3.1. Tan Delta Before PD Activities

**Figure 3:**
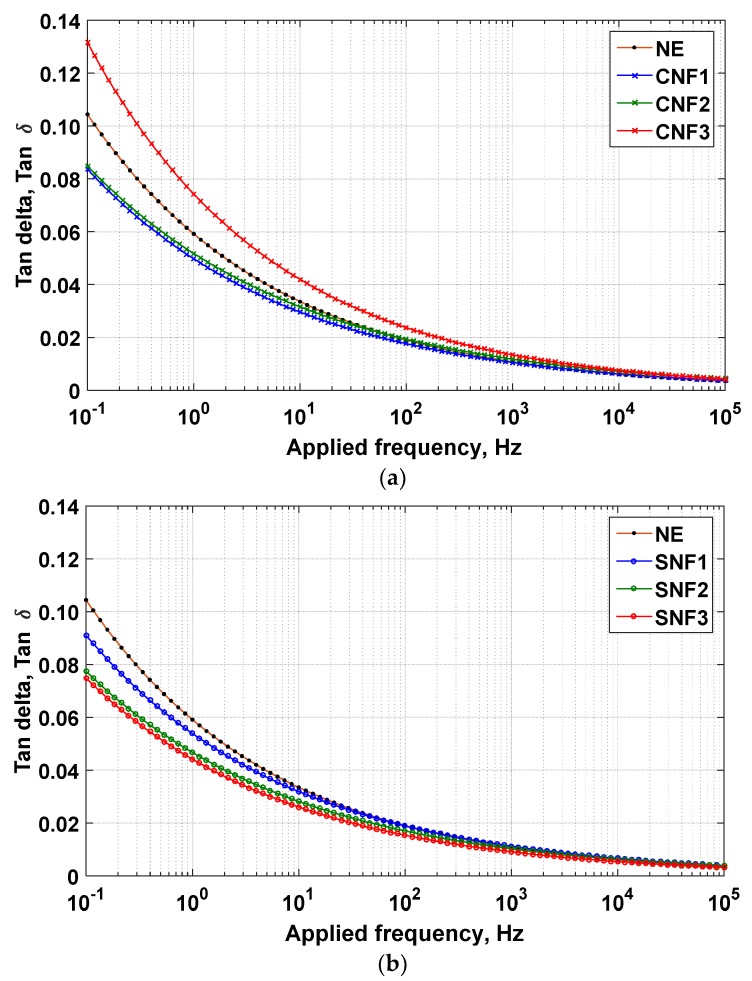
Tan delta as a function of applied frequency for (**a**) CNF and (**b**) SNF samples.

The results of tan delta measurement as a function of frequency and nanoparticle concentration are shown in Figure 3. At lower frequencies (0.1/1.0/10 Hz), higher variation in the tan delta measurements can be clearly seen. This is possibly due to Maxwell-Wagner-Sillars (MWS) interfacial polarization, where the frequency-dependent contributes to the formation of the charge build-up at the nanoparticle interfaces and dipole molecules of the base oil [16]. At higher frequencies (1 k/10 k/0.1 M Hz), it can be seen that the tan delta is strongly influenced by the increment of the frequencies, where the tan delta drops below 0.02 for all samples. 

While investigating the influence of nanoparticles in the base oil at power frequency (50/60 Hz), it is noteworthy that the tan delta peak decreases with the addition of conductive and semiconductive nanoparticles, except for Fe_3_O_4_ at 1.0 g/L. The reduction in tan delta values is associated with the restriction on the mobility imposed by the interaction between nanoparticle electron trapping capabilities and the base oil molecules [17]. When considering the effect of nanoparticle concentration, the tan delta response of the natural ester with conductive nanoparticles (CNF) appears to differ from that associated with the natural ester with semiconductive nanoparticles (SNF). For CNFs, the increasing concentrations up to 1.0 g/L results in a tube-tube conduction mechanism between the particles. This condition is associated with high surface conductivity of Fe_3_O_4_ and particle concentration. As the volume fraction is increased as shown in Figure 3a, tan delta becomes higher due to the increasing interfacial charge conductivity as obtained experimentally.

More importantly, the presence of high amount of conductive nanoparticles in a base fluid means a greater likelihood of tube–tube conduction than in the case of low conductive particles, which refers to TiO_2_ in this instance. With the presence of TiO_2_, there is a slight decrement of tan delta towards the increment of TiO_2_ concentration, such that it still remains less than the base oil, as shown in Figure 3b. This could be due to the reflection of low interfacial charge conductivity upon the increment of TiO_2_ concentration, which subsequently affects the tan delta value. When the content of nanoparticles is increased, it is more difficult to achieve good dispersion of nanoparticles throughout the suspension and aggregation can occur. This results in different interphase conductivity regions, which causes the increment or decrement of tan delta values with increasing nanoparticle concentration.

It is noteworthy that the addition of Fe_3_O_4_ or TiO_2_ in a certain amount (0.01/0.1/1.0 g/L) to base oil occasionally resulted in a small difference in the tan delta response of nanofluids, as can be seen in CNF1-CNF2 and SNF2-SNF3. Although this effect could be associated with the introduction of nanoparticles itself as described previously, as with such cases, it may be suggested that the observed responses are due to the presence of the polar species [18]. With an increasing or decreasing number of nanoparticles, more or less surface hydroxyl groups will present at the interfaces, resulting in a direct consequence on the tan delta values. 

### 3.2. PD Activities at Inception Stage

**Figure 4:**
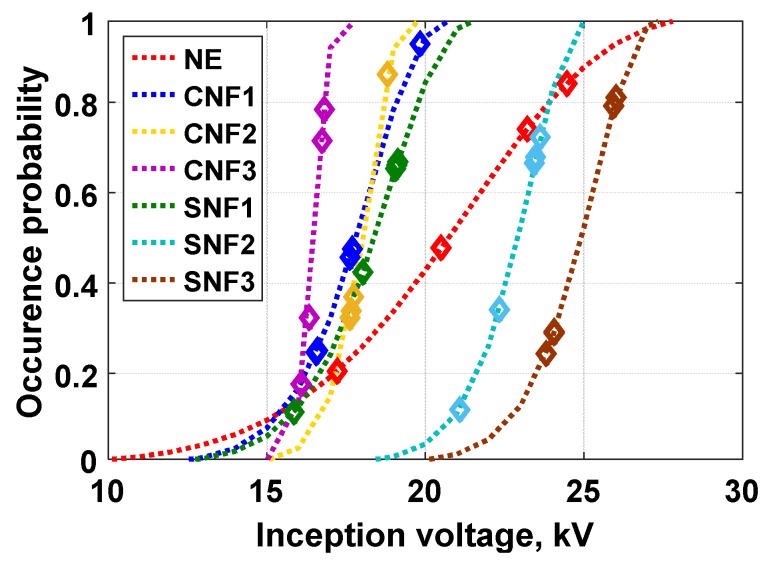
Weibull distribution of PDIV average values for base oil and nanofluid samples.

Partial discharge inception voltage (PDIV) was recorded at the inception condition of the PD measurement when the applied voltage magnitude was increased until the first 100 pC of PD occurred, based on IEC 61294. An occurrence probability distribution of PDIV average results according to Weibull statistical function, which was plotted for each of sample, is shown in Figure 4. Since the lowest inception voltage at which PD occurs is also the main interest for insulation applications, it is important to observe the lower tail of the Weibull distribution slope. Table 2 shows the Weibull parameters for each sample, where α or the scale parameter is the PDIV at 60% of cumulative probability, β is the shape parameter, which indicates the range of PDIV values within the distribution, and $U_{1\%}$ is the prediction of PDIV at 1% of probability.

In general, the shape parameter β of all nanofluid samples is higher than the base oil. This indicates that the incorporation of nanoparticles leads to the narrower distribution of PDIV, or in other words, the localization of PD. A very high localization of PD occurrences is exhibited by the sample with the highest conductive nanoparticle concentration, namely CNF3. The cumulative probability α shows that the incremental increase in nanoparticle concentration contributes to a reduction in PDIV for CNF samples, but there is a significant escalation for SNF nanofluids. At the lowest probability of the inception condition $U_{1\%}$, all nanofluid samples, especially SNF3, have a higher PD tolerance compared to the base oil. This is due to the trapping characteristics of nanoparticles on the charge mobility for both types of nanofluids. Similar observations were also reported by Cavallini et al. in [19].

### 3.3. PD Activities at Steady State Condition

#### 3.3.1. Effect of Nanoparticles Type

**Figure 5:**
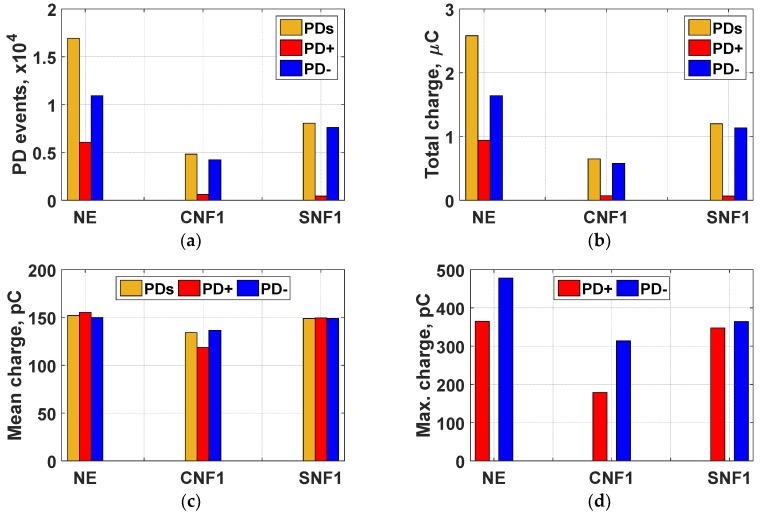
PD measurement results at 26 kVrms in 1500 cycles; (**a**) PD events, (**b**) total charge, (**c**) mean charge, and (**d**) maximum charge magnitude.

The PD measurement results for NE, CNF1, and SNF1 at a steady state of 26 kVrms of applied voltage are shown in Figure 5. With the addition of Fe_3_O_4_ and TiO_2_ nanoparticles, the number of PD events, total charge, mean charge, and maximum charge magnitude are lower compared to the base oil. A comparison between conductive and semiconductive-based nanofluids with equal nanoparticle concentration (0.01 g/L), clearly indicates that the addition of small amounts of conductive nanoparticles, such as Fe_3_O_4_, enhances the PD resistance of nanofluids more effectively than the semiconductive-based TiO_2_.

To explain the differences between the two types of nanoparticles, according to Sima et al., under the applied electric field, both are induced and polarized within a very short timescale [13]. The electrons initiated by charge injection move quickly towards the positively-charged nanoparticle surface. This process represents the electron catchment on the nanoparticle surface and reduces the electron movement and PD activities. Upon completion of the charge catchment, spherical nanoparticles are saturated with negative charges, and thereby no longer trap the electron. The value of charge saturation for each type of nanoparticle is determined by the value of their conductivity and permittivity, where Fe_3_O_4_ nanoparticles can potentially trap higher number of electrons compared to TiO_2_.

**Figure 6:**
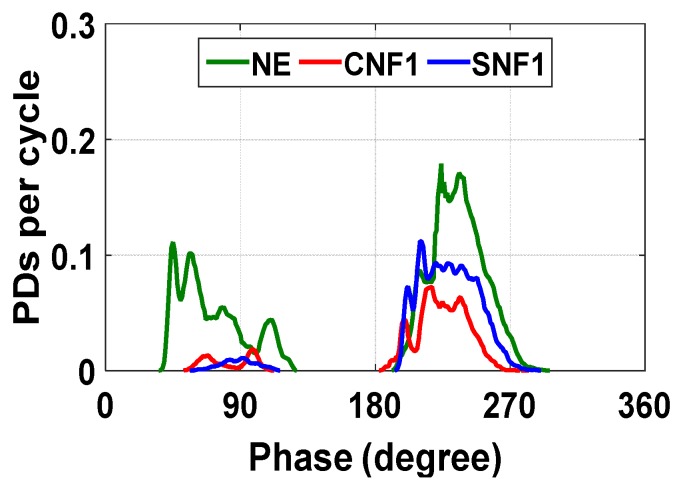
PD phase distribution as a function of nanoparticle type.

Figure 6 presents the PD phase distributions of NE, CNF1, and SNF1 nanofluids. The PD phase distribution shows that most PDs occur from 45° to 113° and 180° to 270°. The peak of the distribution is slightly higher at 180° to 270° for each sample. This is due to the influence of the larger area of the ground electrode in the ionization process during the charge injection. The molecular ionization, which causes the PD occurrences, is dependent on the electric field and geometry of the electrodes. In this mechanism, a high electric field derives a free electron from a neutral molecule and generates a positive ion. The electrons with high mobility relative to positive ions are swept away from the ionization zone and are absorbed by the positive electrode and initiate PDs [11]. In the case of nanofluids, the charging of nanoparticles takes place, during which many of the mobile free electrons produced by the ionization are trapped before they can reach the electrode.

#### 3.3.2. Effect of Applied Voltage

**Figure 7:**
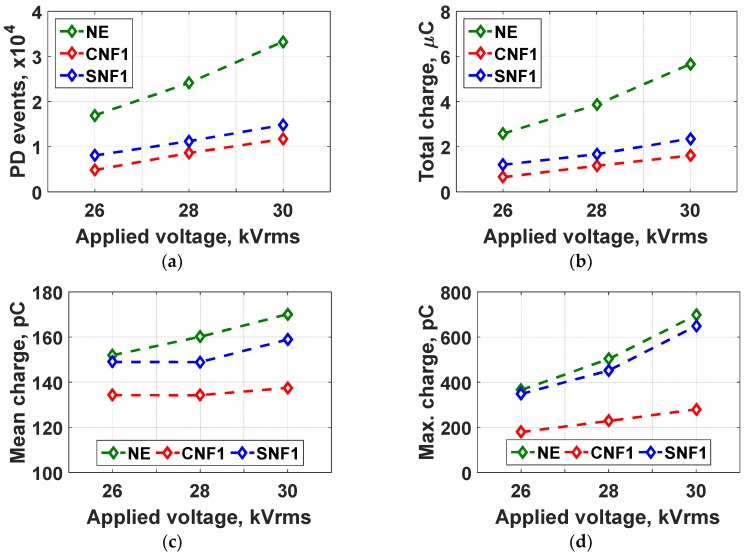
PD activities as a function of the applied voltage amplitudes in 1500 cycles; (**a**) PD events, (**b**) total charge, (**c**) mean charge, and (**d**) maximum charge magnitude.

The PD results obtained for NE, CNF1, and SNF1 as a function of 50 Hz applied voltage amplitude is shown in Figure 7. With increased voltage, the PD events, total charge, mean charge, and maximum charge magnitude also increase, but the mean charge for CNF1 is almost constant. When the applied voltage is increased, the electric field is also increased and the ionization process is enhanced, increasing the free electron generation rate. At this stage, PD events are boosted almost linearly with the applied voltage. The total charge also increases with additional applied voltage due to higher PD occurrences [20]. The mean charge magnitude increases as the applied voltage is increased. Furthermore, the maximum charge magnitude increases with additional applied voltage because higher applied voltage magnitude provides a larger voltage drop across the electrodes when a PD occurs. In the case of nanofluids, both show a lower number of PD activities compared to the base oil at different applied voltage amplitudes.

#### 3.3.3. Effect of Particles Weight Fraction

**Figure 8:**
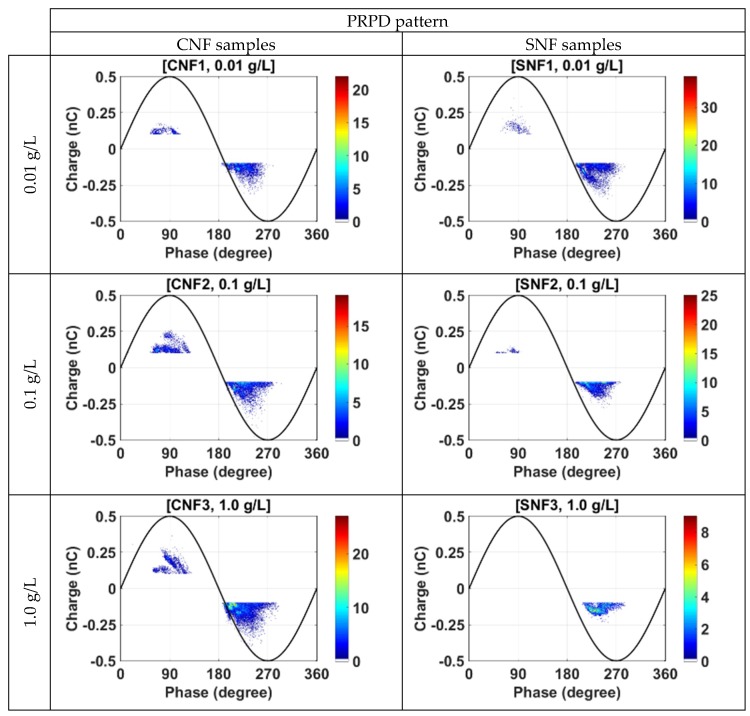
PRPD patterns for nanofluid samples with conductive and semiconductive nanoparticles at 26 kVrms applied voltage in 1500 cycles.

The phase-resolved partial discharge (PRPD) patterns obtained from the experiments are presented in Figure 8. It can be seen that PDs usually occur between 45° and 100° in the positive cycle and between 180° and 270° in the negative cycle. Since the electron generation rate is enhanced by the larger surface area of the ground electrode, this causes more PDs to occur during the negative cycle. Referring to CNF samples, the intensity and magnitude of PDs increase significantly with increasing concentrations of conductive nanoparticles (CNF1, CNF2, and CNF3 samples). Previous research reported that the magnitude and repetition rates of PDs are slightly higher for Fe_3_O_4_ compared to semiconductive and insulating nanoparticle types [19]. Increasing Fe_3_O_4_ concentration up to a certain ratio will also increase the electric dipole-dipole interaction between particles and support their agglomeration and conduction. This consequently leads to the electric field increment between the electrodes, increasing the PD numbers and magnitude.

For SNF samples, the number of PDs and their magnitude decrease with the increasing concentration of semiconductive nanoparticles (SNF1, SNF2, and SNF3). Under certain conditions, such as 1.0 g/L of TiO_2_ concentration, no clear PD activities was obtained from the measurement in the positive cycle. This could be due to the increase in resistance against PDs when TiO_2_ nanoparticles were added to the suspension. Increasing TiO_2_ concentration will also increase dipole–dipole interaction between particles and support the agglomeration process. However, due to the low conductivity of the TiO_2_ nanoparticle surface, it leads to insulation mechanism rather than conduction and trapping of the charge with increasing electron trap density [21]. In contrast, the reduction of PD activities due to the existence of nanoparticles could be beneficial to the power transformer design, where it can reduce its size and space for liquid insulation. 

## 4. Discussion

Under applied high voltage magnitude, the electric field is the main contribution to the ionization process, which leads to PD occurrences and breakdown when the insulation material exceeds the tolerance limit. However, the addition of nanoparticles into the base material modifies the electric field distribution between the electrodes, thus enhancing the insulation performance. 

**Figure 9:**
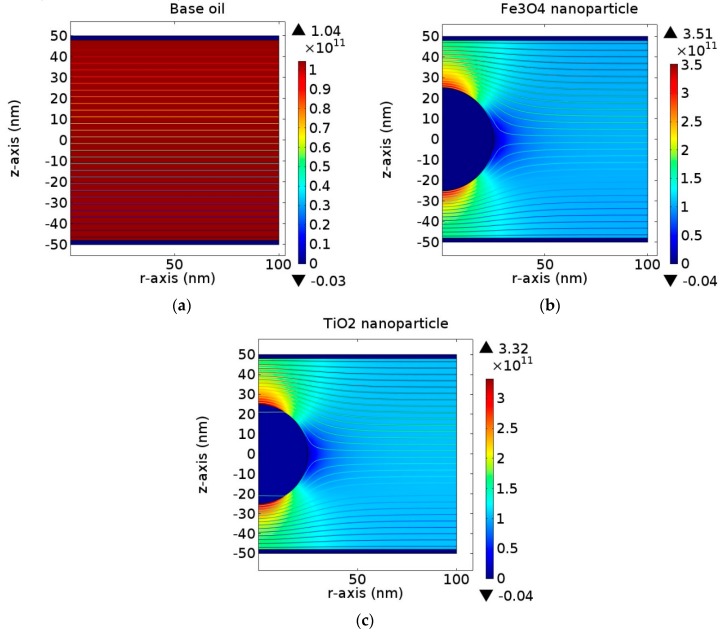
Electric field (V/m) distribution and electric equipotential lines for (**a**) base oil, (**b**) with Fe_3_O_4_ and (**c**) TiO_2_ nanoparticle.

In order to understand the enhancement mechanism in this study, a Finite Element Analysis (FEA) model was developed. The simulated electric equipotential lines and the electric field distribution from the FEA model, with and without nanoparticle (Fe_3_O_4_ and TiO_2_), are shown in Figure 9. The model consists of a spherical nanoparticle of 50 nm diameter suspended in a natural ester oil, which is immersed between two-plane electrode gap of 100 nm under 50 Hz, 10 kV AC applied voltage. The conductivity values of Fe_3_O_4_ and TiO_2_ were set as 1 × 10^5^ and 1 × 10^−11^ S/m, respectively, while the permittivity values were set as 80 and 100 according to the literature [22]. The results are similar with the previous simulation results obtained from a model of a spherical nanoparticle suspended in transformer oil with various permittivity values [23]. Other published research has also used FEA software to obtain the distribution of electric field within nanofluid during streamer discharges [24,25].

**Figure 10:**
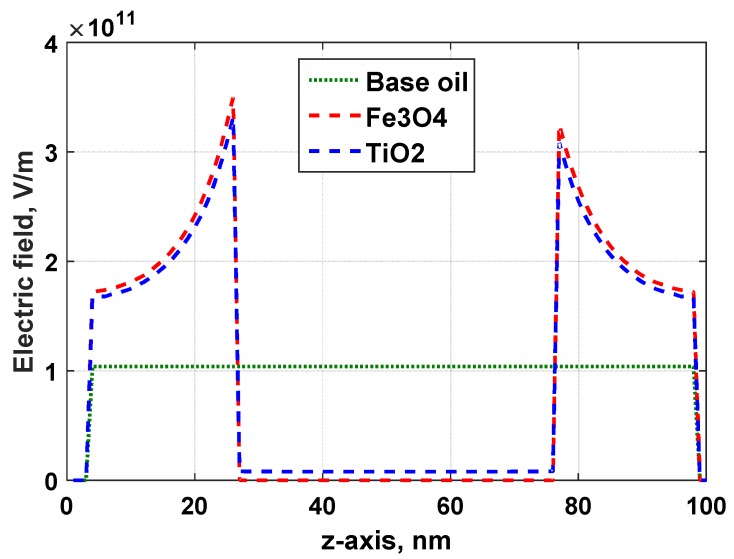
Cross section plot of the electric field magnitude from the FEA model in Figure 9.

In Figure 9a, the electric field of the base oil and surrounding area between the electrodes system is uniformly distributed, since the electrical properties of the dielectric are homogenously constant. The maximum value of the electric field is 1.04 × 10^11^ V/m. When a nanoparticle was added in the oil, the electric field is significantly altered, as shown in Figure 9b,c. Due to the charging dynamic effect, suspending a nanoparticle into base oil results in a higher deformation of the electric field distribution at the particle-oil interface. This increment can be seen clearly along the z-axis of the cross section plot of the electric field magnitude, as shown in Figure 10. The maximum electric field induces or polarizes the electric double layer of conductive particles and semiconductive particles in a very short timescale. This generates positive charges in the upper hemisphere and negative charges in the lower hemisphere, according to the electric field direction. As a result, free electrons initiated from the surroundings due to ionization or charge injection move quickly and are trapped in the positively-charged hemisphere until fully saturated [11]. Fe_3_O_4_ experiences a higher increment of the electric field magnitude. Hence, it highly induces positive charges and traps more free electrons than TiO_2_.

In general, partial discharge (PD) happens once the electric field stress reaches a certain value, which is the inception field. To explain this mechanism in nanofluids, two conditions are considered: (1) inception and (2) steady-state.

**Figure 11:**
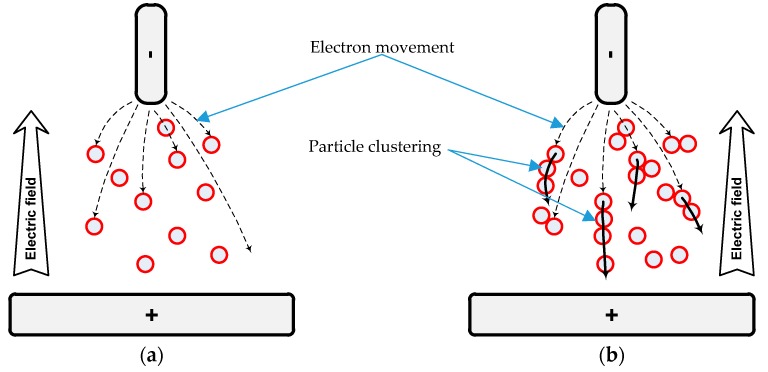
Schematic diagram of electron movement for nanofluids between two electrodes (**a**) at inception condition and (**b**) steady state condition.

PD inception voltage is usually recorded as the applied voltage at which the first PD occurs. It is also considered as a precursor to the incipient dielectric fault of insulation material [26]. This initiation process is random and strongly governed by the local electric field deployed within the electrode-material-electrode system. In the case of nanofluids, the presence of nanoparticles modifies the degree of heterogeneity of the electric field in the nanofluids, which depends on the nanoparticle properties and their dispersion in the base oil [27]. Local intensity of the electric field can result in localized PD occurrences at the same ionization level and applied voltage. As a result, the fluid interphases near the electrode ionize high-mobility electrons and low-mobility ions. Therefore, under the influence of electric field force, they are set up to migrate between two electrode polarities. At such conditions, the nanoparticles begin to act as electrons and negative ion scavengers, which generate a potential well, as depicted in Figure 11a [15].

At PD steady-state condition, assuming the nanoparticles are fully attached and charge-saturated, charges will drive the nanoparticles to move towards the opposite electrode at a very low speed. This is due to dielectrophoretic forces [28], which are responsible for the formation of particle clustering, as illustrated in Figure 11b. This mechanism accelerates the aggregation of nanoparticles along with PD activities, according to their respective concentration levels. For instance, the increase of Fe_3_O_4_ over a certain amount could cause higher PD activities, hypothetically due to the easy formation of conduction interphases. Within a high electric field, accumulated conductive interphase could be converted into a micro-bridging conductive channel [29] and are efficiently utilised by rapid streamer propagation, consequently leading to breakdown [30]. This is the main disadvantage of conductive nanoparticles compared to semiconductive or insulating nanoparticles especially in higher concentrations in base oil.

The results of tan delta and PD activities of natural ester-based nanofluid insulation under the presence of Fe_3_O_4_ and TiO_2_ are inter-connected. It is well known that tan delta can be used as an efficient tool to assess the insulation performance of the insulating materials [31]. The presence of nanoparticles in the base oil has a significant effect on the tan delta of natural ester. In the case of CNF, the nanofluid is sensitive to polarization and may enhance the tan delta. This can also explain why PD can occur easily in CNF compared to SNF. Higher tan delta and lower PD resistance of nanofluids are consistent with solid nanodielectrics, as reported by previous researchers [6], who describe the inter-relationship between the insulation property under low electric field (tan delta) and high electric field (partial discharge). More importantly, the tan delta and PD pattern for different nanoparticle types and concentrations are the findings that most need to be addressed. They could potentially influence the insulation performance when nanofluids are applied in a power apparatus in the future.

## 5. Conclusions

Partial discharge (PD) activities in natural ester-based nanofluid have profound characteristics, according to nanoparticle types and concentrations. The results obtained in this work indicate that nanostructuration significantly improves the dielectric strength of insulating oil in low amounts of both nanoparticle types (0.01 g/L). Consequently, the tan delta is decreased and PD resistance is enhanced with the appropriate amount of nanoparticles. However, a high concentration of conductive nanoparticles, such as Fe_3_O_4_, can increase tan delta value, promoting the PD activities and aggregation process simultaneously.

Since the influence of dielectrophoretic forces on natural ester with conductive nanoparticles (CNF) is more vigorous compared to natural ester with semiconductive nanoparticles (SNF), in terms of formation of interphase conduction as a result of PD activities, it is reasonable to suggest that SNF is more suitable to be applied as an insulation material in high voltage power equipment. 

## Figures and Tables

**Table 1:** Weight fraction of nanofluid formulation samples.

Sample Abbreviation	Amount of Nanoparticles [g/L]	
	Fe_3_O_4_	TiO_2_
NE	-	-
CNF1	0.01	-
CNF2	0.1	-
CNF3	1.0	-
SNF1	-	0.01
SNF2	-	0.1
SNF3	-	1.0

**Table 2:** Weibull parameters of the average partial discharge inception voltages.

Samples	Shape Parameter,β	PDIV Average Value at 60%, α (kV)	PDIV Withstand Value, $U_{1\%}$ (kV)
NE	5.95	22.05	10.18
CNF1	12.40	18.35	12.66
CNF2	21.49	18.30	15.20
CNF3	47.14	16.67	15.12
SNF1	11.77	18.97	12.83
SNF2	20.15	23.34	18.58
SNF3	20.21	25.37	20.21
